# Reimbursement policy optimization for Angiotensin-converting enzyme (ACE) inhibitors in Bulgaria: Controlling expenditure without undermining access to treatment

**DOI:** 10.1186/2052-3211-8-S1-P17

**Published:** 2015-10-05

**Authors:** Rossen M Kazakov, Penka I Petrova

**Affiliations:** 1 BGPharmA, Sofia, 1463, Bulgaria; 2 WBW, Sofia, 1231, Bulgaria

## Background

A reimbursement policy for angiotensin-converting enzyme (ACE) inhibitors based only on controlling expenditure and not adequate for the patient access to treatment could not return expected results for long-term improvement of patients' health. The study addresses the need for optimization of the reimbursement policy of the National Health Insurance Fund in Bulgaria (NHIF) which is based on payment of a certain percentage of the reference price within an INN group of drugs, i.e. 25%, 50%, 75% or 100% and covers the Bulgarian reimbursement market of ACE inhibitors, related to pharmaceutical expenditure and doctors and patients behaviour.

## Methods

The study design is related to policy evaluation and impact assessment of alternative/what-if policy decisions related to reimbursement policy optimization. The methodology employed is mathematical modelling and simulation of the ACE inhibitor drugs market with the aid of computer modelling and simulation software. Designing and testing a reimbursement policy based on lower rates of patient co-payment, while at the same time providing means for controlling pharmaceutical expenditure, is the focus of this study. The simulation experiments use INN/ACE inhibitor prescription data by the NHIF and market data by IMS Health. These data are analysed and then used in a system dynamics model accounting for the doctors prescribing behavior, patient flows and NHIF expenditure, after which a number of policy experimentations are conducted related to alternative policy scenarios. The what-if scenarios are performed using an interactive learning environment or the so-called "management-flight simulator" which enables experimentation through instant changes in independent variables and their effect on the dependent variables within the modelled dynamic system in a historical and forecasted time frame between 2010 and 2020.

## Results

The results show that lowering the level of patient co-payment by raising the level of reimbursement, coupled with incentives to improve access to therapy and compliance, would increase NHIF pharmaceutical expenditure on one hand, but on the other would increase the number of treated patients and at the same time would provide future savings from hospitalization of potential non-compliant and non-treated patients with chronic cardiac disease and cardiac incidents. Designing optimal reimbursement policy related to ACE inhibitors is a highly sophisticated process that needs to account for the dynamic interrelationships among all key independent and dependent factors within a systemic perspective, i.e. reimbursement levels, patient co-payment, access to treatment, compliance to therapy, doctors prescribing behaviour, pharmaceutical expenditure and government incentives to improve healthcare results.

